# Exploring the Experience of Novelty When Viewing Creative Adverts: An ERP Study

**DOI:** 10.3389/fpsyg.2018.00471

**Published:** 2018-04-09

**Authors:** Shujin Zhou, Yue Yin, Tingting Yu, Edward J. N. Stupple, Junlong Luo

**Affiliations:** ^1^Department of Psychology, Shanghai Normal University, Shanghai, China; ^2^Centre for Psychological Research, University of Derby, Derby, United Kingdom

**Keywords:** creative advertising, novelty, ERPs, N2, N400

## Abstract

The electrophysiological correlates of experiencing novelty in creative advertising were studied in 28 healthy subjects using event-related potentials. Participants viewed images that were difficult to interpret until a description was presented providing either a creative description (CD) featuring an unexpected description of the image based on the original advertisement, or a normal description (ND), which was a literal description of the image (and served as a baseline condition). Participants evaluated the level of creativity of the description. The results showed that the N2 amplitude was higher for CDs than for NDs across middle and right scalp regions between 240 and 270 ms, most likely reflecting conflict detection. Moreover, CDs demonstrated greater N400 than NDs in a time window between 380 and 500 ms, it is argued that this reflects semantic integration. The present study investigates the electrophysiological correlates of experiencing novelty in advertising with ecologically valid stimuli. This substantially extends the findings of earlier laboratory studies with more artificial stimuli.

## Introduction

Creativity plays an important role in many aspects of our lives: it is essential in education, arts, science (Fink et al., 2010), and of course in the design and development of creative advertising campaigns (Sasser and Koslow, 2008; Baack et al., 2016). Advertising practitioners consider creativity as an effective solution of breaking through the advertising clutter in a competitive media marketplace (e.g., Ang et al., 2007; Smith et al., 2008; Shirkhodaee and Rezaee, 2014) and it is typical for introductory texts to dedicate a substantial section to the role of creativity in advertising (e.g., Smith and Yang, 2004). It is of theoretical and practical importance to research creativity in advertising. The present study, therefore, investigates creative advertising in an attempt to conduct an ecologically valid examination of the cognitive processing of creativity. For this reason, our stimuli are devised to be more similar to the creative adverts we routinely encounter than standard laboratory tasks for measuring creativity.

Creativity is defined as that which is novel and useful (Sternberg and Lubart, 1993; Fink et al., 2010; Luo et al., 2013). Although there is no consensus on the definition of creativity in advertising, there are two major dimensions for creative advertising on which most researchers agree: divergence and relevance (Smith and Yang, 2004; Smith et al., 2007; Shirkhodaee and Rezaee, 2014; Chen et al., 2016). It is, however, important to bear in mind that the novelty and the usefulness (commonly used in the mainstream creativity research) are synonyms to the divergence and the relevance in the creative advertising research (Ang et al., 2007; Kilgour and Koslow, 2009; Runco and Jaeger, 2012). The first and most fundamental dimension is divergence, which has been studied primarily in creative advertising research (e.g., Smith and Yang, 2004; Smith et al., 2007). It should be noted that divergence here is what distinguishes creative advertising from other standard advertisements. As such, we focused on exploring the novelty (the indicator of divergence) in present research.

A review of the existing empirical research indicates that the research methods of advertising creativity rely primarily on questionnaire investigations (e.g., Smith et al., 2007; Ahmad and Mahmood, 2011; Shirkhodaee and Rezaee, 2014; Chen et al., 2016). There is, furthermore, no literature on creative advertising using neuroscientific methods such as electroencephalography (EEG), functional magnetic resonance imaging (fMRI), and positron emission tomography (PET). EEG provides a temporal resolution of cognitive processes and therefore presents the best method for investigating the time course of electrophysiological processes in this domain. Importantly, the EEG is suggested to diversify methodological approaches in creative advertisement research of novelty.

In contrast to the literature on creativity in advertising, there are numerous electrophysiological studies of creativity. Using EEG, the vast majority of studies report that creative task performance is associated with alpha power changes (Arden et al., 2010). There are, however, a range of different findings in the literature on evoked EEG [or event-related potentials (ERPs)] of creativity, perhaps due to wide variety of creativity tasks used (Luo et al., 2013). The most consistent result among these studies is a positive correlation between creativity (such as insight problem solving) and a N400-like component (Sprugnoli et al., 2017). Insight problem solving was a process of reconstructing the problem in a novel way as an “Aha” experience (Qiu et al., 2008). In a riddle task (Mai et al., 2004), the participants were first presented with the question “The thing that is very old, but very valuable,” then the answer is “antique.” It was found that a negativity (N380) is associated with insight. They speculated that N380 might be N2 or N400. Specifically, participants were proposed to first form a mental set, in order to answer, however, a novel “script” needed to be elicited that conflicted with their expectations. N380 is probably similar with a N2 component and reflects cognitive conflict in breaking an old mental set. Additionally, the authors also proposed that the N380 may be the N400 because the negativity is related to semantic integration. Other negative deflections, for example, N320 (Qiu et al., 2006), were considered to be related to the N2. While N300–500 (Luo et al., 2011) and N430–500 (Luo et al., 2013) have been reported as the N400. Moving toward the late negative component (LNC), a negativity in 2000–2500 ms observed by Qiu et al. (2008) is considered to be a marker of “Aha” feeling. This flash of insight is proposed to be implicated when comprehend the novelty in a creative advertisement. A study of the electrophysiological correlates of the experience of novelty could provide empirical evidence for this proposal.

In the present study, we adapted real examples of creative advertisements and devised two different conditions by changing the advert’s literal description. More specifically, creative adverts’ images were followed by two different types of descriptions: the creative description (CD) and normal description (ND). In the CD condition, participants were presented with an unexpected description, which featured novel description. In contrast, the ND condition presented a literal description of the image and served as a baseline condition. Consequently, the underlying cognitive processing of novelty when viewing creative adverts can be isolated by subtracting the neural activities elicited by ND condition from activities elicited by CD condition. Based on the above discussion and given the existing electrophysiological studies of creativity (Mai et al., 2004; Luo et al., 2013; Sprugnoli et al., 2017), we hypothesized that CD stimuli should be more difficult for participants to understand compared with the ND stimuli on account of the conflict between the initial expectation and the CD. And it seems reasonable to predict that this will result in two processes of novelty, conflict detection, and the integration of semantic information which is incongruent with initial semantic expectancy (Mai et al., 2004; Shen et al., 2017). The process of conflict detection will elicit activation primarily associated with the conflict monitoring (e.g., N2 effect) (e.g., Folstein and Petten, 2008; Enriquez-Geppert et al., 2010). While the process of the semantic integration may be reflected in the N400-like effect that was found to be sensitive to the integration between the retrieved semantic memories and the unrelated concepts, leading to conceptual inference (e.g., Arzouan et al., 2007; Rutter et al., 2012; Rataj et al., 2018).

## Materials and Methods

### Participants

Twenty-eight right-handed healthy volunteers were recruited by advertising in Shanghai Normal University (14 females and 14 males, mean age = 23.23, *SD* = 2.04) and received a financial compensation for participating in the study. No participants had a history of current or past neurological or psychiatric illness and all had normal or corrected-to-normal vision. This study was approved by the local Ethics Committee of Shanghai Normal University, and all participants signed an informed consent form before the experiment.

### Stimuli

**FIGURE 1:**
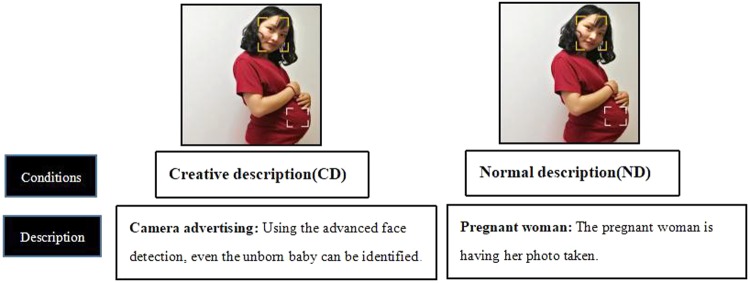
Example of the stimuli. Under the same image, a description was presented providing either (*CD*) a novel description which featured unexpected perspective of the image based on the original advertisement, or (*ND*) a literal description of physical information about the image (and served as a baseline condition).

This study used creative adverts, chosen from the overseas media (e.g., Google, YouTube, and Twitter) to eliminate the possibility of familiarity biases (Sheinin et al., 2011). Original brand logos were omitted to avoid the negative effects of brand familiarity (Shirkhodaee and Rezaee, 2014). Two different types of description (CD and ND) in Mandarin Chinese were developed for each image as the conditions of the present study. The CD was adapted from the original creative advert’s slogan. Some minor changes were made to the descriptions of CD stimuli to make them more accessible to Chinese participants. The ND was developed by removing the CD and replacing it with a description of the available physical information for the image (**Figure 1**).

All the images, 300 × 300 pixel, were color images and presented in the center of the screen. All the descriptions were checked for word length and frequency of occurrence in the Chinese language. There were no significant differences in word length (mean: CD = 12.16, ND = 11.87; *t*_(99)_ = 0.89, *p* = 0.373) and word frequency (mean: CD = 174880, ND = 157960; *t*_(99)_ = 0.94; *p* = 0.347) between the two conditions. Frequency of occurrence in modern Chinese was computed using the online Chinese text computing resource^1^.

To ensure the effectiveness of the stimuli, 144 images along with two kinds of descriptions were pilot-tested with 24 individuals (15 females and 9 males, mean age = 23.54, *SD* = 1.61) who did not participate in the formal ERP experiment. Participants were asked to judge whether these stimuli were creative or not. One of the defining features of creativity in advertising is whether it is surprising (a synonym for unexpected), or nonobvious (Simonton, 2016). Participants were thus instructed to judge the stimuli as creative if they thought the description was unexpected, and as not if they thought the description was expected. The chosen 100 images were selected as the most frequently rated as creative or non-creative. Every CD stimuli was rated as creative and every ND was rated as non-creative, by at least 20 participants.

### Procedure

Participants were seated approximately 110 cm from the screen during the experiment and were familiarized with the procedure and pace of the experimental task during eight training trials. None of the CD or ND trials in the formal experiment were used in the familiarization phase. Two-hundred trials (100 CD stimuli and 100 ND stimuli) were presented randomly without repetition in an event-related paradigm. These were divided into four blocks, participants could rest between each block for at least 30 s. And participants were instructed to try to avoid movement and blinking as much as possible during the presentation of the experimental materials.

**FIGURE 2:**
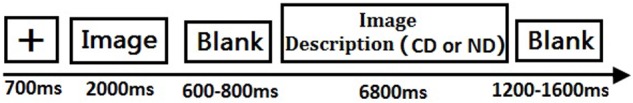
Illustration of an experimental trial.

The experimental paradigm is illustrated in **Figure 2**. On each trial, after the fixation point (+) appeared for 700 ms in the center of the screen, the ad’s image was shown alone for 2000 ms. Then after a jitter of 600–800 ms, either a creative or a ND was displayed below the image for up to 6800 ms or until a response key was pressed. The participants were instructed to rate the description for the given image on a creativity scale of 1–3 before the description disappeared. If participants thought that the description for the given image was normal and expected, they rated the description as non-creative by pressing key “1”; if participants thought the description was novel and unexpected, they rated the description as “creative” by pressing key “2”; if participants thought the description was very novel and very unexpected, they rated the description as “very creative” by pressing key “3.” The reason why we set up the 3-point scale is to explore whether the neural correlates of novelty is modulated by different levels of evaluation. Participants were instructed to respond as quickly as possible. The trials in which participants pressed the other keys (e.g., “0” and “4”), or pressed the key “1” in CD, or pressed the key “2” or “3” in ND were considered invalid. Trials were separated by a 1200–1600 ms blank interval.

### ERP Recording

The electroencephalogram signals were continuously recorded from 64 Ag/AgCl electrodes sewn into an elastic cap (NeuroScan, Inc., United States), arranged according to the International 10–20 System, with the reference on the left mastoid. To monitor eye movements and blinks, the horizontal electrooculogram (EOG) was recorded from electrodes placed on the bilateral external canthi and the vertical EOG was recorded from electrodes placed on the supraorbital and infraorbital ridges of the left eye. All interelectrode impedance was kept below 5 kΩ. Both EEG and EOG were continuously sampled at 500 Hz, with a 0.01–100 Hz bandpass using the NeuroScan Synamps 2 digital amplifier system (Neuroscan Labs, El Paso, TX, United States). Ocular artifacts were rejected offline. Single trials were rejected when the response was improper or contaminated by blinks, eye movements, and excessive muscle activity (voltage exceeded ± 100 in any channel).

### ERP Data Analysis and Statistics

In the present study, the ERP waveforms were time locked from the onset of the stimuli (image + description) to the response press action. Epochs began 200 ms prestimulus baseline in each trial and continued for 1000 ms afterward. The ERP waves under each condition were obtained after the ERP of the two types of descriptions were overlapped and averaged, respectively. Only those CD trials which the participants indicated to be creative by pressing “2” or “3” key were taken into account for the ERP analysis. Likewise, only those ND trials which the participants indicated to be non-creative by pressing “1” key were taken in account for the ERP analysis.

**FIGURE 3:**
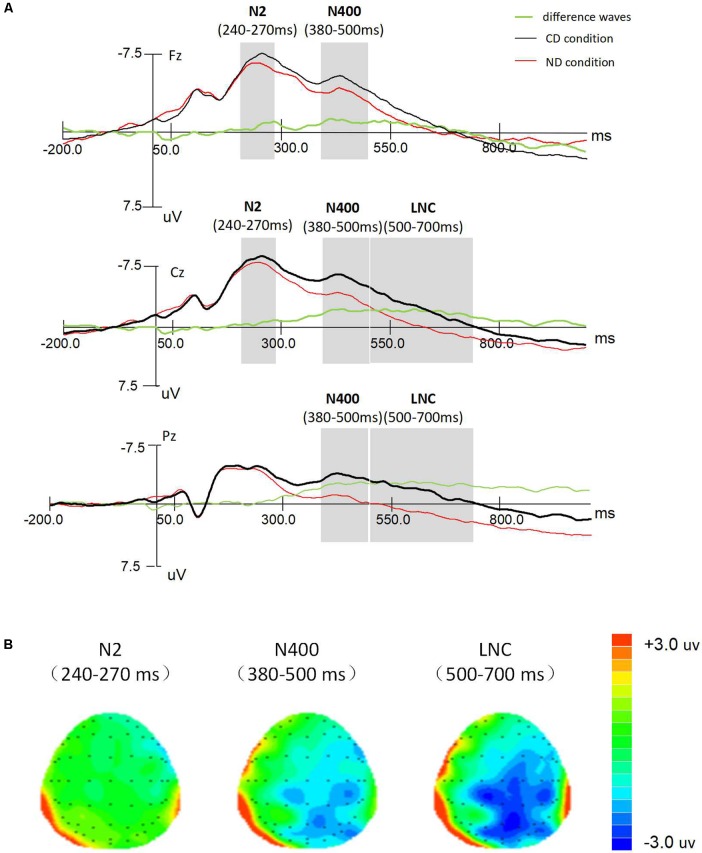
**(A)** Grand average ERPs at Fz, Cz, and Pz for two conditions (CD and ND) and difference waves (CD–ND). **(B)** Topographic map for CD condition versus ND condition difference wave in the time range 240–270, 380–500 and 500–700 ms.

Based on the grand-averaged waveform (**Figure 3**), the N2 occurred from 240 to 270 ms, and the N400 occurred from 380 to 500 ms. The mean amplitudes of both the N2 (between 240 and 270 ms) and the N400 (between 380 and 500 ms) were measured relative to baseline. In addition, the mean amplitude of the LNC was assessed in the time window between 500 and 700 ms. As shown in the topographies map (**Figure 3**), the N2 component was mainly distributed over the anterior regions, while the amplitude of N400 and LNC primarily distributed over the posterior scalp. In addition, according to the preexisting studies (Mai et al., 2004; Luo et al., 2011, 2013), 21 electrodes were chosen for statistical analysis: AF3, AF4, FPz, F3, F4, Fz, FC3, FC4, FCz, C3, C4, Cz, CP3, CP4, CPz, P3, P4, Pz, PO3, PO4, POz. Thus, for the N2 component, a 2 (CD/ND) × 3 (left: AF3, F3, FC3, and C3; middle: FPz, Fz, FCz, and Cz; right: AF4, F4, FC4, and C4) × 4 (anterior: AF3, FPz, and AF4; frontal: F3, Fz, and F4; frontocentral: FC3, FCz, and FC4; central: C3, Cz, and C4) three-way repeated measures ANOVA was conducted. For the N400 component, like the LNC component, a 2 (CD/ND) × 3 (left: AF3, F3, FC3, C3, CP3, P3 and PO3; middle: FPz, Fz, FCz, Cz, CPz, Pz, and POz; right: AF4, F4, FC4, C4, CP4, P4, and PO4) × 7 (anterior-frontal: AF3, FPz, and AF4; frontal: F3, Fz, and F4; frontocentral: FC3, FCz, and FC4; central: C3, Cz, and C4; centroparietal: CP3, CPz, and CP4; parietal: P3, Pz, and P4; parietal-occipital: PO3, POz, and PO4) three-way repeated measures ANOVA was conducted. SPSS 16.0 was used to conduct repeated measures ANOVA. For all analyses, a threshold significance criterion of *p* < 0.05 was used. When the factors did not meet the sphericity assumption, the Greenhouse–Geisser correction was used. *Post hoc* analyses were conducted using Bonferroni corrected *t*-tests.

## Results

### Behavioral Performance

The average rate of key “1” responses to the 100 ND trails was 93.7 ± 5.7% and the average rate of key “2” and key “3” responses to the 100 CD trails was 46.3 ± 23.0 and 32.6 ± 26.1%, respectively, and the mean reaction times (RTs) to the stimuli for the three response types were 1585 ± 468.35, 2908 ± 705.30, and 2775 ± 872.57 ms, respectively (**Table 1**). Indeed, there is a minor issue such that there is an imbalance in the rate of three response buttons (keys “1,” “2,” and “3”). The influence of this could be examined in a future study. A one-way (very creative/creative/normal) repeated measures ANOVA on average rate and RTs yielded significant main effects of the three response types for average rate, *F*(1.14, 30.77) = 52.83, *p* < 0.001, $\eta_{p}^{2}$ = 0.66 and RTs, *F*(1.83, 49.35) = 104.95, *p* < 0.001, $\eta_{p}^{2}$ = 0.80. The results indicated that mean RTs were shorter for the baseline condition than for the creative condition (*p* < 0.001) and for the very creative condition (*p* < 0.001), However, there was no significant difference between the creative stimuli and the very creative stimuli (key “2” versus key “3”) in the RTs (*p* = 0.510), likewise, in the average rate (*p* = 0.354).

**Table 1:** Mean RTs and rate (*M* ± *SD*) for three response types (very creative/creative/normal).

	Key “1”	Key “2”	Key “3”
RTs (ms)	1585 ± 468.35	2908 ± 705.30	2775 ± 872.57
Rate (%)	93.7 ± 5.7	46.3 ± 23.0	32.6 ± 26.1

The one-way (CD/ND) repeated measures ANOVA on the mean RTs revealed that the significant main effect among the two types of description, *F*(1.00, 27.00) = 181.75; *p* < 0.001; partial eta squared $\eta_{p}^{2}$ = 0.871. Specially, the mean RT of ND (*M* = 1585 ms, *SD* = 468.35) was faster than that of CD (*M* = 2812 ms, *SD* = 725.40).

### ERP Results

The ERP grand-averaged waveforms (**Figure 3**) showed that creative stimuli elicited a more negative ERP deflection than normal stimuli in the time interval between 240 and 270 ms. Then, in the time interval between 380 and 500 ms, creative stimuli elicited a more negative ERP deflection than normal stimuli. Finally, creative stimuli elicited a more negative ERP deflection during 500–700 ms time window. Mean amplitudes in the time windows of 240–270, 380–500, and 500–700 ms were analyzed using repeated-measures ANOVAs. Note that there was no significant difference for the three components between creative stimuli by pressing key “2” and that of very creative stimuli by pressing key “3” [N2: *F*(1.00, 27.00) = 0.31, *p* = 0.588, $\eta_{p}^{2}$ = 0.01; N400: *F*(1.00, 27.00) = 1.10, *p* = 0.302, $\eta_{p}^{2}$ = 0.04; LNC: *F*(1.00, 27.00) = 1.10, *p* = 0.302, $\eta_{p}^{2}$ = 0.04]. The results clearly demonstrate that the neural basis of novelty was not sensitive to levels of evaluation.

For the mean amplitude of N2, there was a marginal significant main effect of description type, *F*(1.00, 27.00) = 2.94, *p* = 0.098, $\eta_{p}^{2}$ = 0.09. Pairwise comparison of the main effect of description type showed that the N2 mean amplitude between 240 and 270 ms of creative stimuli was marginally more negative than that of normal stimuli. In addition, the main effect of hemisphere site was significant, *F*(1.39, 37.76) = 13.93, *p* < 0.001, $\eta_{p}^{2}$ = 0.34, reflecting that the N2 component was larger across middle and right hemisphere sites. The main effect of antero-posterior site was significant, *F*(1.63, 44.89) = 8.40, *p* < 0.01, $\eta_{p}^{2}$ = 0.23, reflecting that the N2 component was largest at anterior locations. There was also a significant description type × hemisphere interaction [*F*(1.39, 36.14) = 12.95, *p* < 0.001, $\eta_{p}^{2}$ = 0.32]. None of the other main effects or interactions was significant. To further explore the interaction effect, *post hoc t*-tests revealed that the N2 mean amplitude was more negative for creative stimuli than that for normal at right hemisphere [*t*(111) = -5.72, *p* < 0.001, *d* = 5.26] and at midline hemisphere [*t*(111) = -3.36, *p* < 0.001, *d* = 3.18].

Analyses of the N400 component showed significant main effects of description type [*F*(1.00, 27.00) = 17.62, *p* < 0.001, $\eta_{p}^{2}$ = 0.40], hemisphere site [*F*(1.52, 40.97) = 23.53, *p* < 0.001, $\eta_{p}^{2}$ = 0.47], and antero-posterior site [*F*(1.34, 36.26) = 50.61, *p* < 0.001, $\eta_{p}^{2}$ = 0.65]. There was also a significant description type × hemisphere × antero-posterior interaction effect [*F*(4.05, 109.28) = 4.33, *p* < 0.01, $\eta_{p}^{2}$ = 0.14]. *Post hoc t*-tests which were conducted to break down this interaction effect showed that the N400 mean amplitude was more negative for creative stimuli than that for normal stimuli at right hemisphere [*t*(83) = -11.26, *p* < 0.001, *d* = 1.23] and midline hemisphere [*t*(83) = -10.23, *p* < 0.001, *d* = 1.11] over centroparietal scalp regions.

For the LNC during 500–700 ms, a three-way repeated-measures ANOVA demonstrated that the creative stimuli elicited marginally greater LNC amplitude than the normal stimuli, *F*(1.00, 27.00) = 3.83, *p* = 0.061, $\eta_{p}^{2}$ = 0.12. There were significant main effects of hemisphere site [*F*(1.70, 45.99) = 27.60, *p* < 0.001, $\eta_{p}^{2}$ = 0.51] and antero-posterior site [*F*(1.44, 38.76) = 16.60, *p* < 0.001, $\eta_{p}^{2}$ = 0.38]. And there was a significant description type × hemisphere × antero-posterior interaction [*F*(4.59, 124.05) = 2.64, *p* < 0.05, $\eta_{p}^{2}$ = 0.09]. *Post hoc t*-tests showed that in the right hemisphere [*t*(83) = -5.521, *p* < 0.001, *d* = 0.60] and the midline hemisphere [*t*(83) = -5.84, *p* < 0.001, *d* = 0.64] creative stimuli elicited more negative LNC than normal stimuli over the posterior scalp.

## Discussion

The behavioral data showed that participants responded faster in the ND condition than in the CD condition, indicating that creative advertising, compared with the normal stimuli, was more difficult to process. This was perhaps due to the need for greater cognitive effort to interpret the novelty of the creative advertising stimuli which that automatic or routine processes may not be able to interpret (Donkers and van Boxtel, 2004). The question of how the property of novelty was interpreted can be elucidated more clearly based on the ERP data.

As predicted, the N2 (240–270 ms) and N400 (380–500 ms) were observed with larger amplitudes in the CD condition than that in the ND condition. A greater LNC (500–700 ms) was moreover detected in the creative stimuli than normal stimuli. For the difference wave (CD–ND), the N2 mean amplitude elicited greater negativity activity over right and midline hemisphere scalp regions; the N400 mean amplitude was distributed with strong negative activity over right hemisphere and the most posterior scalp regions; the amplitude of LNC was mainly distributed over the posterior scalp.

In comparison with the normal stimuli, at right and midline hemisphere, the N2 effects elicited by the incongruous CD stimuli may indicate conflict detection processes (Folstein and Petten, 2008; Enriquez-Geppert et al., 2010). In the current study, the conflict in the creative stimuli may indicate an incongruity between the initial expectation and the CD. This may be because the initial expectation of the image based more on the physical information about the image while the CD interprets the image from a novel perspective. Novelty in creative advertising allows participants to change perspective, such that participants may have certain expectations when scanning the image before the presence of the description; if the description then presents no conflict with expectation, the participants can follow automatically and respond quickly without surprise or confusion. However, participants are likely to feel puzzled or confused when there is a conflict with their initial expectation, and at that point they detect and try to resolve the incongruence.

The N400 effects induced by the text in the CD condition compared with the baseline condition were most likely linked to the semantic integration stage which was where we propose that the processing of conflict between the initial expectation and the CD occurred. Only participants who understand the incongruity are likely to make favorable evaluations of the creativity of the stimuli (Shirkhodaee and Rezaee, 2014). As Mai et al. (2004) summarized, N400 is associated with the processing of semantic information that is incongruous with semantic expectancy by retrieving semantic concepts from long-term memory (Mai et al., 2004). In the present study, participants were required to detect the conflict and then integrate it into their mental set by finding a link between the image and its description. To succeed, participants have to integrate information from semantic memories with unrelated information in the current context in order to achieve conceptual inference (Arzouan et al., 2007; Rutter et al., 2012; Rataj et al., 2018). Once the inference had been made, they would elicit a novel “script” that was different from their expectation so as to form a new interpretation matching with the image (Mai et al., 2004).

The increase in the LNC amplitudes in response to CD suggesting the possibility of a positive emotional response that is expected to accompany the moment of insight induced by forming novel associations (Sprugnoli et al., 2017). The possibility that the primary distinguishing feature of the stimuli could be humor (which can often be the result of novelty) is a factor that should be pursued in future work to unpack the roles of these different aspects of creative advertising. Alternatively, this finding is nicely in line with the recent work by Rataj et al. (2018). The reduction in positivity for CD compared to ND might be explained as the overlap between the delayed negative effects stemmed from the N400 and the LPC (Rataj et al., 2018). This overlapping might be related to higher working memory that plays a role in the construction of new association in understanding CD (Rataj et al., 2018). That is, the more positive LPC expected for CD, the more overlap by late negativity, and thus the more reduction in the amplitude of LPC (Arzouan et al., 2007; Rataj et al., 2018). At the same time, there is no need to map novel association for ND, thus appearing as increased amplitude of LPC (Rataj et al., 2018).

In short, our results provide electrophysiological correlates of experiencing novelty in advertising with higher ecological validity compared to other laboratory studies with more artificial stimuli. Such that, the novelty of creative advertising – the conflict between the initial expectation and the presented text – can be monitored or detected, as revealed by the N2 effect. In addition, as indicated by the N400 effect, the novelty of creative advertising is understood by retrieving semantic concepts from memory and generating a novel connection between the image and the CD. These findings not only demonstrate that the neural correlates of creative thinking generalize to more “real-world” tasks, but also that a neural process akin to insight occurs when we view creative advertisements.

## Author Contributions

JL and SZ designed this study. SZ, YY, and TY performed the study. JL and SZ analyzed the data. SZ drafted the manuscript. JL and ES reviewed the manuscript.

## Conflict of Interest Statement

The authors declare that the research was conducted in the absence of any commercial or financial relationships that could be construed as a potential conflict of interest.

## Footnotes

1 http://lingua.mtsu.edu/chinese-computing/
